# Comparison of Trap and Equine Attraction to Mosquitoes

**DOI:** 10.3390/insects14040374

**Published:** 2023-04-11

**Authors:** Sarah C. Dilling, Saundra H. TenBroeck, Jerome A. Hogsette, Daniel L. Kline

**Affiliations:** 1 Department of Animal Sciences, University of Florida, Gainesville, FL 32611, USA; 2 USDA–ARS, Center for Medical, Agricultural and Veterinary Entomology, 1600 SW 23rd Drive, Gainesville, FL 32608, USA

**Keywords:** vacuum aspirators, mosquitoes per horse, CDC 1012 trap, Mosquito Magnet-Pro trap

## Abstract

**Simple Summary:**

Mosquitoes are pests of horses. We evaluated the ability of traps to protect horses from these blood feeders. To evaluate comparative attraction, a horse was placed 3.5 m from a mosquito trap. Fewer mosquitoes were trapped because mosquitoes were more attracted to the horse. Trapped mosquitoes increased after removing the horse from the area. Increasing trap attraction by adding horse odors into the trap airstream was unsuccessful because the horse supplying the odors could not be moved far enough away. Three mosquito traps spaced at the study site showed that mosquitoes were not evenly distributed and fewer were collected near structures. This shows the importance of trap placement. When horses were vacuumed, 324 and 359 mosquitoes/h were found feeding on the animals during summer and fall studies, respectively. A nearby mosquito trap simultaneously captured 224 mosquitoes/h. The numbers of mosquitoes vacuumed simultaneously from two horses showed that one horse attracted twice as many mosquitoes as the other. All horses are not equally attractive to mosquitoes. For better mosquito management with traps, the attractive range of traps and host animals must be known. Traps must be as attractive to mosquitoes as host animals so traps can better protect host animals.

**Abstract:**

Mosquitoes are pests of horses, but mosquito trap efficacy data, especially the ability of traps to protect horses, are lacking. Studies were conducted to investigate the comparative attraction between traps and horses, increase trap attraction by adding horse odors to the airstream of a trap, determine the spatial distribution of adult mosquitoes, estimate the numbers of mosquitoes feeding on horses, determine the relative attraction of horses to mosquitoes, and estimate the range of mosquitoes’ attraction between two horses. When a horse and a mosquito trap were placed 3.5 m apart, there was a significant reduction in mosquitoes entering the trap. Adding horse odors to the airstream of a trap produced equivocal results because the horse providing the odors influenced the trap catches. Mosquitoes were not evenly distributed across the study site, which emphasized the importance of trap placement. Vacuuming mosquitoes from the horses in different seasons demonstrated that 324 and 359 mosquitoes per hour were feeding during the two studies. Separate analysis of data from the two horses vacuumed simultaneously revealed that one horse attracted twice as many mosquitoes as the other. This caused the results of a study to determine the attraction range of two horses moved from 3.5 to 20.4 m apart to be inconclusive.

## 1. Introduction

Biting flies are known to feed on horses and cause characteristic defensive behaviors [1] as the animals try to avoid the painful bites [2]. Many chemical products are sold for use by horse owners to counter the effects of mosquitoes and other biting flies [3]. However, the overuse of some products has resulted in reduced efficacy and the development of pesticide resistance [4]. Repellents have been developed for use on horses [5], but most publications relate to human subjects [6]. It has been suggested that traps can be beneficial for mosquito management because of the variety of traps and baits [7].

Mosquito traps are considered to be very effective as surveillance tools for monitoring the seasonal prevalence and species composition of mosquitoes in a specific area [8,9]. Traps can also be effective in reducing mosquito numbers in the trap vicinity [10]. Many different mosquito traps, varying greatly in effectiveness and usefulness, have been developed [11]. Many commercial traps are advertised to control mosquitoes on one acre of property and are commonly used around equine and other livestock facilities. Most of these traps use attractive stimuli associated with a host animal, such as heat, carbon dioxide (CO_2_), kairomones, and moisture [12]. But how effective are mosquito traps when they are competing with a natural host? Campbell et al. [13] conducted a series of experiments comparing mosquito traps and a horse and found a significant difference between the species and total quantity of mosquitoes collected from mosquito traps compared with mosquitoes vacuumed directly from a horse. This raises the question: Do trap catches provide a true representation of the actual mosquito species in the population? Trap catches could potentially be improved by adding odors directly from horses to the specified trap. Little research has been conducted which directly compares the efficiency of mosquito traps with a natural host in the immediate proximity.

Therefore, the main objectives of the following studies were to (1) conduct competitive trapping studies between traps and horses; (2) evaluate the effects of adding horse odors to the airstream of a trap; (3) compare mosquito location profiles at selected locations at the University of Florida (UF) Horse Teaching Unit (HTU); (4) determine the mosquito numbers and species feeding on horses at selected times; and (5) determine the spacing required between two horses so that they attract mosquitoes as separate entities.

## 2. Materials and Methods

**Figure 1:**
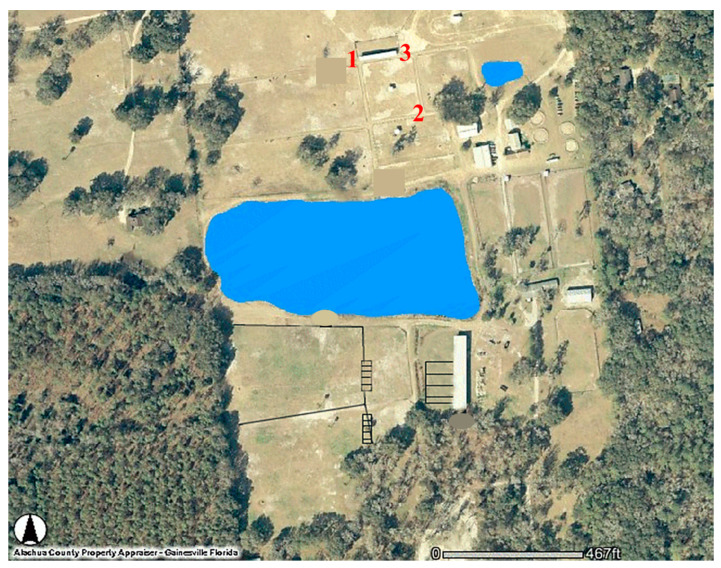
UF Horse Teaching Unit (HTU) showing the placement of the 3 Centers for Disease Control (CDC) 1012 traps near a horse barn in the Location Profile study.

Study site—All studies were conducted in Gainesville, FL, USA, at the UF HTU (Figure 1), located about 4.8 km south of the main campus. 

This 26 ha equine facility housed approximately 40 head of quarter horses varying in sex and age. The site was selected because it is continuously populated by horses and adult mosquito populations are known to be prevalent in high numbers throughout much of the year [13]. Horses at the HTU were maintained by UF staff according to the university’s standards. Because the horses used in our studies were maintained according to university standards, the project did not need approval from a Review Board.

**Figure 2:**
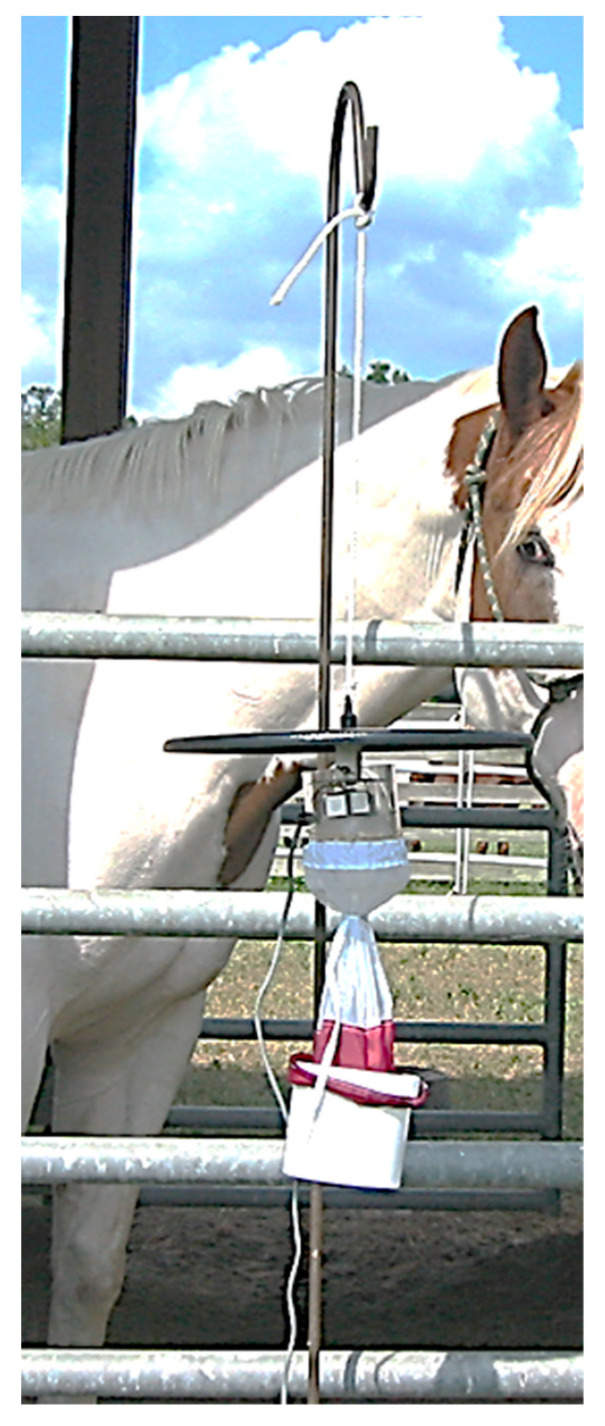
CDC 1012 trap suspended from a metal shepherd’s hook 1 m from the gate of a 3 × 3 m stall.

Mosquito traps and collection devices—The Centers for Disease Control (CDC) trap model 1012 (John W. Hock Company, Gainesville, FL, USA) (Figure 2), with slight modifications, was used during these studies. 

A 6.3 volt light that acts as a visual attractant was eliminated to decrease the number of attractive variables associated with the trap. An in-line fan powered by a 6 volt motorcycle battery, recharged daily, created a suction to capture mosquitoes attracted to the top entrance of the trap beneath the lid and blow them down into a polypropylene collection container with a screened bottom. Unless otherwise stated, in the following studies, carbon dioxide (CO_2_) was supplied by compressed gas cylinders at a regulated rate of 500 mL/min through a Nalgene line that was attached with rubber bands to the side of the trap. The CO_2_ was released near the top entrance of the trap beneath the lid and directly above the level of the in-line fan. 

**Figure 3:**
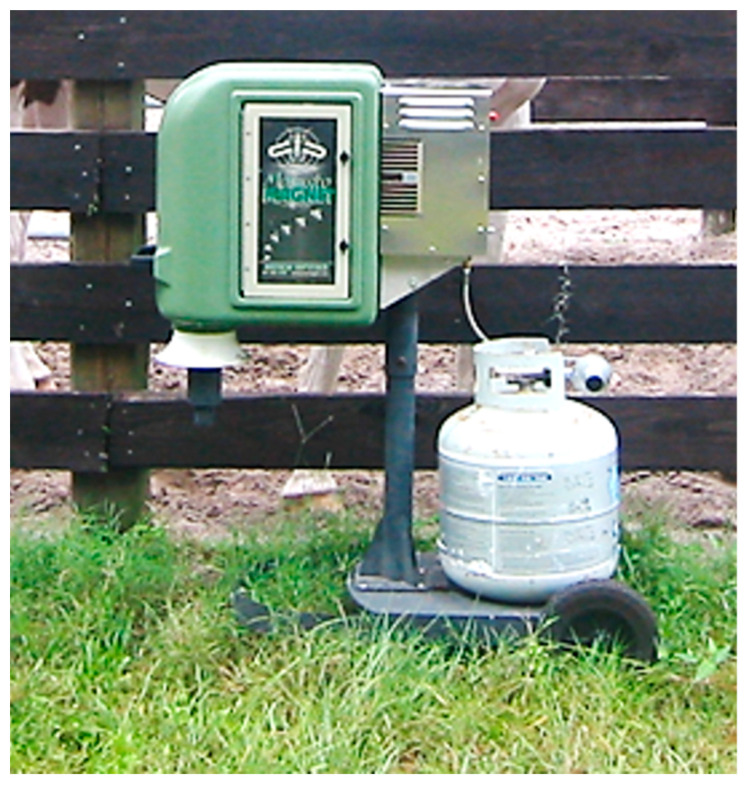
Mosquito Magnet-Pro (MM-Pro) trap placed next to the gate of a 3 × 3 m horse feeding stall.

The Mosquito Magnet-Pro (MM-Pro) (American Biophysics Corporation, North Kingston, RI, USA) (Figure 3) uses patented technology that catalytically converts propane into electricity to power the suction fan, and produces CO_2_, heat and moisture, which attract mosquitoes to the counterflow of the suction fan and released attractants. 

The attracted mosquitoes are sucked into a collection net inside the trap shell where they dehydrate and die. The MM-Pro is 2 m high and constructed of stainless steel with a PVC shell.

**Figure 4:**
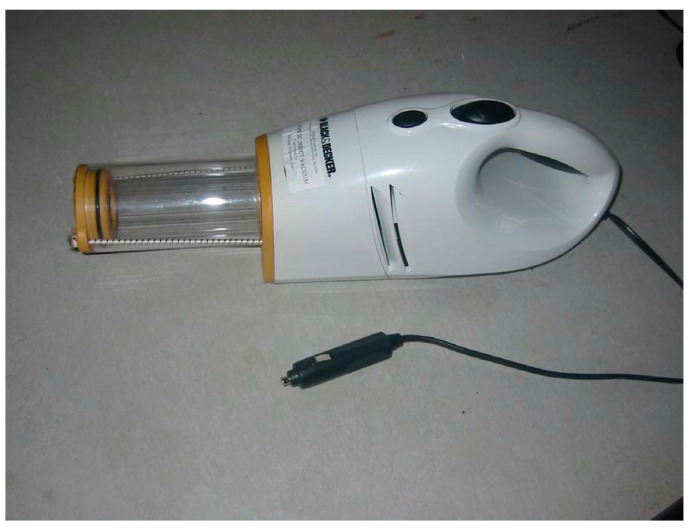
Hand-held portable vacuum aspirator used to collect mosquitoes that landed on the horses.

Mosquitoes that landed on the horses were collected with a hand-held portable vacuum aspirator (DC Insect Vac. BioQuip, Rancho Dominguez, CA, USA) (Figure 4). 

The aspirator was powered by 12 volt AC supplied by an electrical cord with a plug modified to fit into the cigarette lighter outlet of a vehicle. A plastic collection container with a screened bottom was fitted to the vacuum airstream and contained the mosquitoes while the vacuum was turned on. When the vacuum was turned off, the container was quickly sealed with a cap. Horses were acclimated to the aspirator during a 1-day training period.

Horses—A Paint gelding and an Appaloosa mare, both approximately 7 years of age, were used for these studies. The horses were housed at the UF HTU and remained there after the experiment concluded.

Comparative trapping studies—Trapping studies were conducted with the two traps and the Paint gelding described above to evaluate the comparative attraction between the traps and the horse. The experimental designs, similar for both traps, are shown below.

CDC 1012 trapping study—The CDC 1012 comparative trapping study was conducted from 19 July–21 August 2004. Starting 30 min after sunset, a CDC 1012 trap was suspended from a metal shepherd’s hook 1 m from the gate of a 3 × 3 m stall at the end of a single row of similar stalls in a horse barn (Figure 2). Stalls were under a roof but were otherwise open to the environment. No other horses were present in the barn and the treatment horse could not contact the trap. The trap was baited only with CO_2,_ and the collection container was emptied after each of five 20 min trapping periods. There were two treatment groups: no horse present and horse present. During the nights when the treatment horse was to be present, the Paint gelding was placed in the stall adjacent to the trap during the second 20 min trapping period and was then removed from the area. Treatments 1 or 2 for the series of study nights were selected from a pre-determined schedule. Collected mosquitoes were stored at 0 °C until identification and counting were conducted. Each treatment—horse present and no horse present—was repeated six times.

MM-Pro trapping study—The MM-Pro comparative trapping study was conducted from 1–21 October 2004. Starting 30 min after sunset, an MM-Pro trap was placed next to the gate of a 3 × 3 m horse feeding stall at the end of a single row of similar stalls (Figure 3). Stalls were completely open to the environment with no roof. No horses were present in the other stalls and the treatment horse could not contact the trap. The trap was baited with the CO_2_, moisture, and heat that it produced as described above. The collection net was changed after each of five 20 min trapping periods. There were two treatment groups: no horse present and horse present. During the nights when the treatment horse was to be present, the Paint gelding was placed in the stall during the second 20 min trapping period and was then removed. Collected mosquitoes were stored at 0 °C until identification and counting were conducted. Each treatment—horse present and no horse present—was repeated six times.

**Figure 5:**
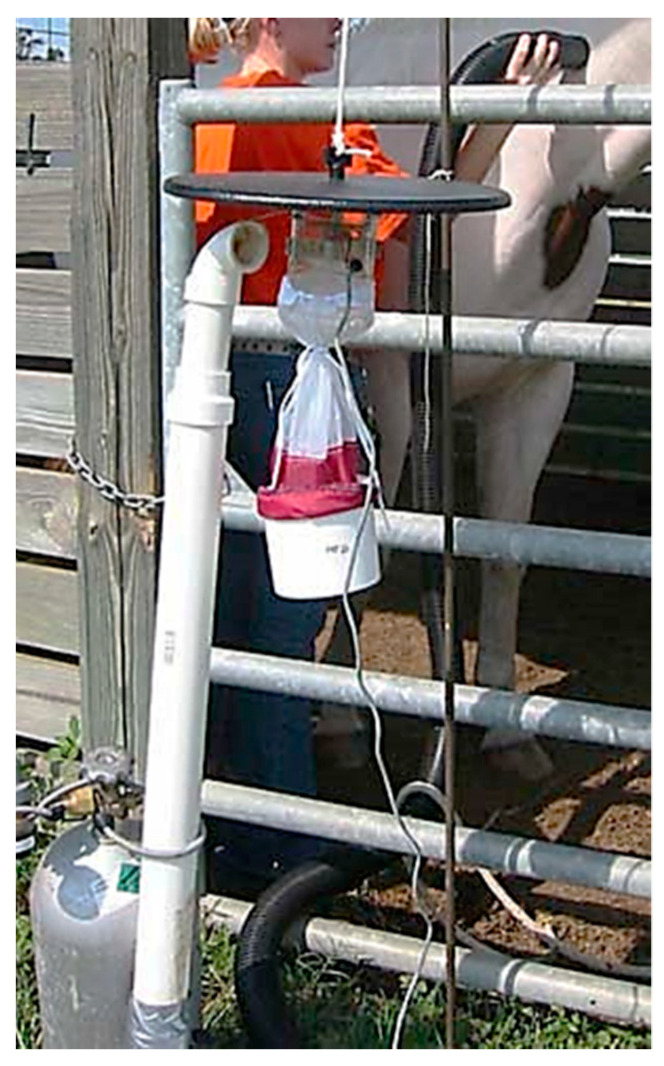
Fabricated vacuum system for adding horse odors to the airstream of a CDC trap.

Horse odor study—This study was conducted from 27 August–24 September 2004 to determine whether the addition of equine odors vacuumed from a horse and added to the airstream of a modified CDC 1012 trap affects the number of mosquitoes captured. A handheld vacuum system was fabricated for this purpose (Figure 5). 

A 2 in diameter, 3 m long flexible, plastic shop vacuum hose was connected to a 2 in diameter polyvinylchloride (PVC) pipe with a 90-degree elbow. The open end of the elbow was connected to a length of 2 in diameter PVC pipe long enough to reach the air intake area near the top of a CDC trap when the pipe was placed vertically on the ground and the CDC 1012 trap was on a shepherd’s hook as described above. A second 2 in diameter 90-degree elbow PVC pipe was placed on the top of the vertical PVC pipe with the open end directed towards the CDC 1012 trap. The vertical PVC pipe was held in place by securing it to the compressed gas cylinder that supplied the CO_2_ for the trap. A battery-powered in-line fan was mounted inside the PVC pipe to create the suction needed to pull air into the shop vacuum hose near the horse and blow it out the top end of the vertically mounted PVC pipe near the trap intake.

Starting 30 min after sunset, a CDC 1012 mosquito trap was placed directly next to the gate of the stall in the horse barn as described above for the CDC 1012 trapping study. An empty collection container was placed on the trap after each of four 20 min trapping periods. For treatment 1, the Paint gelding was maintained in the stall during periods 1, 2, and 3. The CDC trap was baited with CO_2_ only during periods 1, 3, and 4. During period 2, the fan in the PVC pipe was activated so that when the horse was vacuumed with the shop vacuum hose, the CDC 1012 trap became baited with CO_2_ plus the horse odor. The horse was vacuumed all over the body (Figure 5) only during period 2. The horse was removed from the stall at the end of period 3 and trap collection continued for 20 more minutes during period 4. This 4-period protocol was repeated six times.

For treatment 2, also conducted from 27 August–24 September 2004, no horse was present at any time. During periods 1–4, a CDC 1012 trap was set up as described for treatment 1, but the trap was baited only with CO_2_. An empty collection container was placed in the trap after each of four 20 min trapping periods. Collected mosquitoes for both treatments were stored at 0 °C until identification and counting were conducted. Treatments 1 or 2 for the series of study nights were selected from a pre-determined schedule. Because of time constraints, treatment 2 was repeated 4 times.

Location profile study—A mosquito location study using CDC 1012 traps was conducted from 13 July–26 August 2004 to determine the similarity of species captured from different locations at the HTU by the same trap model. Traps were placed at three locations at the UF HTU (Figure 1) and suspended from shepherd’s hooks as described previously. Locations 1 and 3 were 33 m apart at either end of the horse barn described above. Location 2 was 73 and 65 m from locations 1 and 3, respectively, and close to a swampy area. All three traps were baited only with CO_2,_ as described above. The traps were set up 30 min before sunset, i.e., 2000 h, and allowed to collect until mosquito peak activity had concluded, i.e., 2230 h. Mosquitoes were stored at 0 °C until identification and counting were conducted.

**Figure 6:**
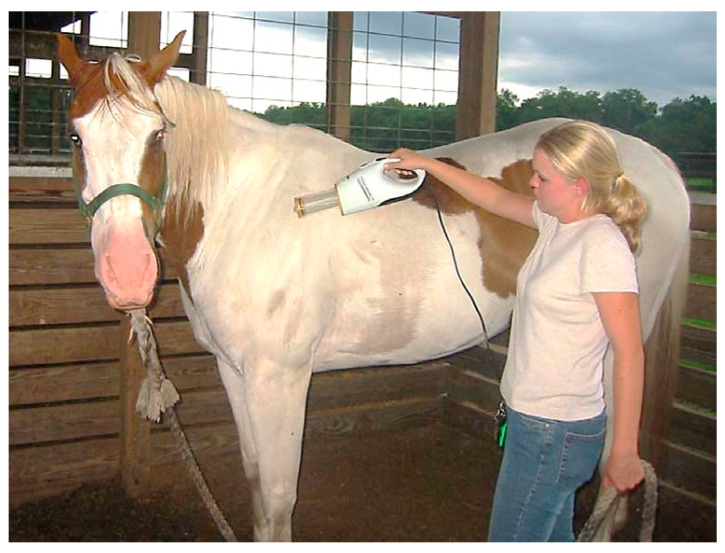
Vacuuming mosquitoes from the Paint gelding using the hand-held portable vacuum aspirator.

Horse vacuuming study—The number of mosquitoes feeding on a horse during a pre-determined period was estimated 6 times between 15 June–12 July 2004 and then repeated 6 times between 4–22 October 2004. The first portion of the study was conducted with the Paint gelding placed in the end stall in the barn described in the MM-Pro trapping study. Mosquitoes were vacuumed from the skin using portable vacuum aspirators (Figure 6) for a 1 h period, starting 40 min after sunset. 

During the second portion of this study, the Appaloosa mare and the Paint gelding were placed in adjacent feeding stalls, described in the MM-Pro trapping study, and vacuumed simultaneously with portable vacuum aspirators for 1 h and 40 min, starting at 30 min after sunset. Mosquitoes were vacuumed from all parts of the bodies of the horses. Vacuumed mosquitoes were stored at 0 °C until identification and counting were conducted. Summer and fall portions of the study were conducted during the time intervals listed above when facilities were available.

Separate entity study—The distance required for the mosquitoes to distinguish two horses as separate entities was examined from 4–22 October 2004. The Paint gelding and the Appaloosa mare were placed in adjacent feeding stalls described in the MM-Pro trapping study. Starting 30 min after sunset, the horses were vacuumed simultaneously for 20 min. The collection containers on the aspirators were then removed and replaced with empty collection containers. The Appaloosa mare remained in the original end stall, while the Paint gelding was moved to another stall depending on the pre-determined assignments for the night. The horses were then vacuumed again for 20 min. This procedure was repeated until the horses were vacuumed in all five stalls. Maximum distances (D) between stall 1, which measured 3.05 m, and the remaining 4 stalls were D2 = 6.09 m, D3 = 9.14 m, D4 = 12.19 m, and D5 = 20.42 m. Captured mosquitoes were stored at 0 °C until identification and counting were conducted.

Data analysis—Count data from the studies described above were analyzed with the General Linear Models (GLM) and Proc Means Programs after transformation with log10 (n + 1) [14]. Although transformed values were used for the analyses, back-transformed values are shown in text and tables. Tukey’s Studentized Range Test [14] was used for separation of the means at a significance level of *p* = 0.05.

## 3. Results

CDC 1012 trapping study—There were 2249 mosquitoes collected by the CDC 1012 trap during this study, with *Mansonia titillans* (Walker) comprising 76% of the total catch, followed in descending order by *Coquillettidia perturbans* (Walker) (11%) and *Culex salinarius* (Coquillett) (7%). When the horse was placed in the stall next to the trap during period 2 of the Horse Present treatment, there was a significant reduction in the mean number of mosquitoes captured compared with periods 1 and 3. This trend was not seen for periods 1–3 in the No Horse Present treatment (Table 1). 

The mean number of mosquitoes captured during period 2 for the No Horse Present treatment was significantly higher than the mean numbers of mosquitoes captured for periods 1 and 3 (Table 1). After period 2, mean numbers of mosquitoes captured in both treatments tended to decrease more during each subsequent period.

MM-Pro trapping study—There were 5533 mosquitoes collected by the MM-Pro trap during this study, with *Cx. nigripalpus* Theobald comprising 36% of the total catch, followed in descending order by *Ma. titillans* (31%) and *Psorophora columbiae* (Dyar and Knab) (23%). When the horse was placed in the stall next to the trap during period 2 of the Horse Present treatment, there was a significant reduction in the mean number of mosquitoes captured compared with periods 1 and 3. This trend was not seen for periods 1–3 in the No Horse Present treatment (Table 2). 

The mean number of mosquitoes captured during period 2 for the No Horse Present treatment was not significantly higher than the mean numbers of mosquitoes captured for periods 1 and 3 (Table 2). After period 2, mean numbers of mosquitoes captured in the No Horse Present treatment tended to decrease more during each subsequent period.

Horse odor study—The total number of mosquitoes collected by the CDC 1012 trap during treatments 1 (Horse Present) and 2 (Horse Never Present) were 1109 and 1671, respectively, with *Ma. titillans* comprising 76% of the total catch, followed by *Cx. nigripalpus* (28%). For treatment 1, there was no significant difference between the mean numbers of mosquitoes captured during periods 1 and 3 (Horse Present) and period 2 (Horse Present plus horse odor vacuumed) (Table 3). 

After the horse was removed from treatment 1, the mean numbers of mosquitoes captured by the trap in period 4 increased significantly (*p* = 0.05). For treatment 2 (Horse Never Present), the mean number of mosquitoes increased significantly from period 1 to period 2, then decreased significantly in periods 3 and 4 (Table 3). This was much the same pattern as was seen in the above CDC1012 and MM-Pro trapping studies when no horse was present (Table 1 and Table 2). 

**Figure 7:**
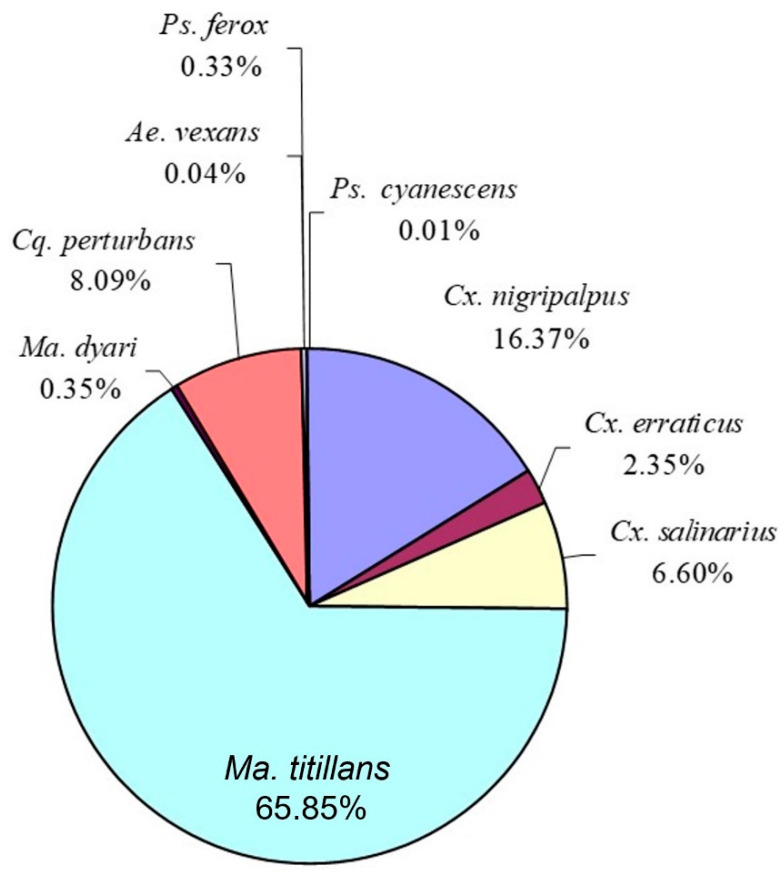
Mosquito species represented as a percent of total mosquitoes collected by three CDC 1012 traps during the location profile study conducted on 13 July–26 August 2004 at the UF HTU, Gainesville, FL, USA.

Location profile study—A total of 7184 mosquitoes was collected by the three CDC 1012 traps during the Location Profile study conducted on 13 July–26 August 2004. The most numerous species was *Ma. titillans*, comprising 66% of the total catch, followed by *Cx. nigripalpus* (16%) (Figure 7). 

Significantly more mosquitoes ($\bar{X}$ = 531.0 ± 188.2) were trapped in location 2 than in locations 1 ($\bar{X}$ = 103.1 ± 36.2) and 3 ($\bar{X}$ = 200.1 ± 59.9), which were not significantly different. The species captured in all three locations were the same but there was a major difference in the percentage catch of two species. In location 1, *Cx. nigripalpus* represented almost 50% of the catch, followed by *Ma. titillans* at 30% (Table 4). However, in locations 2 and 3, *Ma. titillans* represented >70% of the catch, followed by *Cx. nigripalpus* at slightly more than 10% (Table 4).

Horse vacuuming study—The total number of mosquitoes vacuumed from the Paint gelding during the 15 June–12 July 2004 period was 1946, or 324 mosquitoes/h. The total number of mosquitoes vacuumed from the Appaloosa mare and the Paint gelding combined during the 4–22 October 2004 period was 7197 (718 mosquitoes/h or 359 mosquitoes/h/horse), assuming no difference in attraction between horses. During the October study, an MM-Pro trap used for other studies was 27 m distant and in operation during the period when the two horses were vacuumed. The trap captured a total of 2247 mosquitoes, or 224 mosquitoes/h. There were 38% more mosquitoes vacuumed/h/horse than were captured in the trap/h.

During the June–July portion of the study, the most prominent species vacuumed from the horse was *Cq. perturbans*, comprising 39% of the total catch, followed in descending order by *Cx. salinarius* (37%) and *Ma. titillans* (12%) (Table 5). 

During the October portion of the study, the most prominent species vacuumed from both horses combined was *Cx. nigripalpus*, comprising 41% of the total catch, followed in descending order by *Ma. titillans* (27%) and *Ps. columbiae* (17%) (Table 5). We were unable to obtain species data from the MM-Pro trap being used for other studies.

When the total number, number/h, and the species composition of mosquitoes vacuumed from the Appaloosa mare and the Paint gelding were compared separately, the results were quite different. Twice as many mosquitoes/h were vacuumed from the Appaloosa mare (∑ = 4810, or 480 mosquitoes/h) than from the Paint gelding (∑ = 2387, or 238 mosquitoes/h). Approximately 50% of the total mosquitoes vacuumed from the Appaloosa mare were *Cx. nigripalpus*, followed by *Ma. titillans* (21%) and *Ps*. *columbiae* (15%) (Table 5). Approximately 40% of the mosquitoes vacuumed from the Paint gelding were *Ma. titillans*, followed by *Cx. nigripalpus* (25%) and *Ps. columbiae* (21%) (Table 5).

Separate entity study—Mean numbers of mosquitoes vacuumed from the Appaloosa mare were always significantly greater than those vacuumed from the Paint gelding (Table 6).

Species captured from both horses were similar but present in different numbers. For the Appaloosa mare, the species captured were *Cx. nigripalpus* > *Ma. titillans* > *Ps. columbiae*, and for the Paint gelding, the species captured were *Ma. titillans* > *Cx. nigripalpus* > *Ps. columbiae*. For the Appaloosa mare, numbers captured increased during the first two 20 min collection intervals, then began to decrease. For the Paint gelding, the largest number of mosquitoes was captured during the first 20 min interval, then numbers began to decrease.

## 4. Discussion

CDC 1012 and MM-Pro trapping studies—Before our studies were conducted, it was suggested that an equine host placed near a mosquito trap would cause the trap to become more attractive and result in an increase in the trap catch. However, studies 1 and 2 demonstrate that the CDC 1012 and MM-Pro traps captured almost 95% fewer mosquitoes when a natural host was within 4 m.

During the five 20 min trapping periods when no horse was present, there was an increase in the number of mosquitoes trapped during the hour after sunset and a steadily decreasing number trapped during the three remaining periods. This coincides with 2003 studies [15] which demonstrated that most mosquito species have a bimodal flight activity pattern, with a larger peak occurring soon after sunset followed by a smaller peak just prior to dawn. When a horse was added to the system during the second 20 min period of the total 100 min session, the expected increase did not occur. Significantly fewer mosquitoes were trapped, but only when the horse was present. As soon as the horse was removed, the mosquitoes captured in the traps increased significantly. A horse placed next to a trap did not increase the catch; rather, it greatly decreased the catch. In Kenya, mosquito traps captured 98.5% fewer mosquitoes when placed 2.5 m from a human subject in a study designed to use nearby humans to increase trap catches [16]. The equine and human hosts were more attractive to the mosquitoes than the traps.

Data from some studies indicate that the MM-Pro is a superior mosquito trap compared with the CDC 1012 [11,17] but others [13] found that the CDC 1012 trap performed as well or better than the MM-Pro. Thus, we expected both traps to perform similarly; however, neither could compete well when a horse was nearby.

The changes in the species composition between the two studies were most likely seasonal. The CDC 1012 trapping study was conducted during July and August, and the MM-Pro trapping study was conducted in October. The most prominent mosquito species trapped during the CDC 1012 summer trapping study were *Ma. titillans* and *Cq. perturbans*. *Culex salinarius*, a suspected summer vector species at the time, represented only 7% of the catch. In overnight trapping studies [13] in the summer with the CDC 1012, *Cx. salinarius* represented 54% of the total catch with *Ma. titillans* and *Cq. perturbans* being 3% and 2%, respectively. Perhaps larger numbers of *Cx. salinarius* fly later in the night and were thus underrepresented in our shorter studies. There might also have been changes in relative population numbers among the species. During the MM-Pro fall trapping study, the most prominent mosquito species trapped were *Cx. nigripalpus*, *Ma. titillans*, and *Ps. columbiae*. The percentages of *Cx. nigripalpus*, a suspected fall vector species at the time, and *Ma. titillans* were very similar. During overnight studies in the fall with the MM-Pro trap [13], *Cx. nigripalpus* represented almost 90% of the catch. Again, changes may be due to overnight trapping periods or variations in population levels among species.

Data from these studies indicate that trap efficiency is greatly reduced when a host is within 3 m. However, most traps are probably placed more than 3 m from a horse by the layperson, and they have been shown to capture potential vector species [13]. Further research is needed to examine the effects of distance between a live host, e.g., a horse, and a mosquito trap to estimate how far away the trap must be from the host to no longer be negatively affected.

Horse odor study—When the Paint gelding was vacuumed to add its skin odors to the airstream of the CDC 1012 trap, the addition of these odors did not significantly increase the catch. This was possibly because the horse used in the study was too close to and was out-competing the trap. The hose used to transport the odors was 3 m long, but it should have been longer to allow for more separation between the horse and the trap. There was concern about a longer hose affecting the chemical components of the horse odors. Collection and identification of horse and pony skin and hair odors can be difficult [18], and perhaps their effects on the attraction of mosquitoes to traps can be demonstrated empirically with an improved apparatus design.

Location profile study—The location profile study demonstrated that trap placement can be important for catching not only numbers of mosquitoes, but also particular species present in the environment. All three traps captured the same mosquito species but in different percentages. If it can be assumed that the traps attract and capture certain mosquito species that are within an undetermined distance and that mosquito species are attracted similarly to each trap despite its location, then it appears that the mosquito species were not spatially distributed uniformly across the area. Heterogeneous distribution of mosquito species is corroborated by other studies as well [19,20]. In addition, because there were as many as 10 trapping nights during the 6 wk study period, data indicate that the spatial distributions changed very little over time. Because trapping potential vector species is an important factor, results from traps placed in locations 2 and 3 indicated that comparatively few *Cx. nigripalpus* were in the attraction range of those traps. More research is needed to substantiate these findings.

Campbell et al. [13] conducted an overnight trapping study similar to ours at the HTU from July–September 2002. Their traps were rotated through five locations, none of which were as close to a structure as locations 1 and 3 were in our study. For the CDC 1012 trap and four other mosquito traps, at least 86% of the mosquitoes captured were *Cx. nigripalpus* [13]. Each of the other species captured, including *Ma. titillans*, represented <5% of the total catch. Unfortunately, data showing trap catch by location are not available. However, if location data from our location profile study are combined, catch rates of 65 and 17% were recorded for *Ma. titillans* and *Cx. nigripalpus*, respectively. This represents a big disparity between our study in 2004 and that conducted in 2002 [13]. It could indicate a large difference in species population levels between 2002 and 2004.

Traps 1 and 3 were 33 m apart at the opposite ends of a row of 10 stalls open to the environment on the sides but covered by a roof. Stall 1 next to trap location 1 had been enclosed in a white fabric on all four sides from top to bottom for another study. It is possible that this fabric caused a change in the mosquitoes attracted to the trap since mosquitoes have been shown to be attracted or repelled by different colors and light intensities [21]. The trap in location 2, approximately 65 m from the stalls and near a swampy area, captured between 2 and 3 times more mosquitoes, respectively, than the traps in positions 1 and 3 near the stalls. Thus, the stalls appeared to have some effect on mosquito distribution, which was reflected by a decrease in trap catch.

Horse vacuuming study—During the 6 trapping nights in the June–July and October studies, a total of 1946 and 7197 (∑ = 9143) mosquitoes were vacuumed from the horses just during the 1 and 1.67 h sampling intervals, respectively. These numbers should cause concern simply because of the sheer abundance of mosquitoes routinely attracted to and possibly feeding on horses in a short interval at the HTU. The feeding rates were 200–300 mosquitoes/h in the June-July and October studies, respectively, during the hour beginning 30 min after sunset, when mosquito population activity begins to peak [15]. These estimates are probably greatly underestimated depending upon how many mosquitoes visited the horse but escaped prior to capture by the vacuum aspirator.

Large numbers of mosquitoes feeding on horses can have a direct effect on the animals’ health by increasing the chances for pathogen transmission. Indirect nuisance effects can result in wasteful conditions, blood loss, and other health and economic factors, such as reductions in weight gains [22,23], that have been comparatively easy to define in cattle but not in horses. Horses that are too busy fighting off biting mosquitoes may neglect grazing or drinking adequate water. When a UF equine health management class project kept teams of students at the HTU overnight, students noticed that horses in nearby paddocks were rarely at rest (S.H.T., unpublished).

It was noticed during the vacuuming studies that mosquitoes congregated on the horses in preferred landing places. These included the neck, legs, heels, barrel, and stomach. It was interesting to note that the Appaloosa mare always had mosquitoes congregating around her cornet bands; this was not seen with the Paint gelding. Both horses were very quick to shake off the mosquitoes by twitching their skin or knocking them off with their tail. Sometimes the horses dislodged the biting mosquitoes faster than they could be vacuumed.

If traps are being used in a mosquito management system, it would be helpful to know if the traps and the horses are attracting the same mosquito species and whether potential vector species, if present, are well represented. Results from the 19 July–21 August 2004 CDC 1012 trapping study indicated that *Ma. titillans* (76%) was present in large numbers, followed by *Cq. perturbans* (11%) and *Cx. salinarius* (7%). However, during the 15 June–12 July 2004 vacuuming study where mosquitoes were captured directly from the horse, *Cq. perturbans* (39%) and *Cx. salinarius* (37%) were almost equally represented but *Ma. titillans* (12%) was present in much smaller numbers (Table 5). In an April 2003 study at the HTU [3], percentages of *Cx. salinarius* and *Ma. titillans* captured by CDC 1012 traps or vacuumed from a horse were similar. *Cq. perturbans* captures were very low across all traps and a horse.

Results from the 1–21 October 2004 MM-Pro trapping study indicated that the main species present were *Cx. nigripalpus* (36%), *Ma. titillans* (31%), and *Ps. columbiae* (23%). However, during the 4–22 October 2004 portion of the vacuuming study, combined data from the two horses indicated that the main species vacuumed were closely related to the MM-Pro catches: *Cx. nigripalpus* (41%), *Ma. titillans* (27%) and *Ps. columbiae* (17%). When data from the two horses were tabulated separately, results from the Paint gelding, also used in the 15 June–12 July 2004 vacuuming study, were *Ma. titillans* (40%), followed by *Cx. nigripalpus* (25%) and *Ps. columbiae* (21%). Results from the second horse, the Appaloosa mare, were *Cx. nigripalpus* (50%), followed by *Ma. titillans* (21%) and *Ps*. *columbiae* (15%) (Table 5). In a 17 July–30 September 2002 study at the HTU [13], 87% percent of the MM-Pro catch was *Cx. nigripalpus*, and none of the other species exceeded 2.5%. In comparison, on the horse, *Ma. titillans* (40%) was the most numerous species followed by *Cx. nigripalpus* (27%). The percentages of mosquito species captured by traps and vacuumed from horses appear to vary independently over time, and verifying that traps and horses are attracting the same mosquito species would be difficult with the currently available technology.

The difference in attraction between the Paint gelding and Appaloosa mare was completely unexpected and may be the first documented example in the literature. This differential attraction could cause confusion when trying to determine how well the mosquito numbers and species captured by traps compare with the mosquito numbers and species attracted by horses. In our studies, mosquito species vacuumed from the Paint gelding were similar to those captured by the CDC1012 and the MM-Pro traps, but percentages of the species vacuumed from the horse and those captured by the traps were different. This effect was noted in the 17 July–30 September 2002 study [13]. Mosquito species vacuumed from the Appaloosa mare were similar to those captured by the MM-Pro trap, and percentages of the species vacuumed from the horse and those captured by the trap were similar. This effect was also documented in the April 2003 study [13].

The attraction of *Cx. nigripalpus* to the Appaloosa mare appeared to be greater than its attraction to the Paint gelding. The reason is unknown, but it could be a difference in sex or hair color. *Culex nigripalpus* is an opportunistic feeder and shifts its host selection based on the season, feeding on avian hosts in the winter and spring and on mammalian hosts in the summer and fall [24]. It has been stated that male hosts are more attractive to mosquitoes than female hosts [25], but the opposite was seen during this study. More mosquitoes were vacuumed from the Appaloosa mare than from the Paint gelding. Since the male horse was a gelding (i.e., castrated), the lack of testosterone could have affected the mosquito preference. More research is needed to better define the chemicals associated with equine attraction for mosquitoes.

Separate entity study—Ideally, one would expect to see similar mean values from each horse for the number of mosquitoes captured when the horses were close together. This did not happen, even when the horses were only separated by 3.05 m (Table 6). The phenomenon of hosts or traps attracting similar mean numbers of insects when the hosts or traps are close together has been reported for various dipteran species [26]. The means would be expected to vary only after the attractive objects were moved apart enough to no longer interfere with each other’s attractive range [27]. Unfortunately, the means of mosquitoes captured in our study were always significantly different and the attractive range of the horses could not be determined.

One reason for this discrepancy, as was discovered in the vacuuming study above, is that there was an attraction differential between the two horses used in the study. The Appaloosa mare captured nearly twice as many mosquitoes as the Paint gelding and the same mosquito species were attracted in different proportions. If the mean values for mosquitoes captured by the Paint gelding are multiplied by 2, the resulting values are closer to the mean values of mosquitoes captured by the Appaloosa mare (Table 5). However, as the horses were moved farther apart, there was no indication of an onset of independent attraction, e.g., differences between horses in numbers of mosquitoes captured. It was found that mosquito species at HTU are relatively unform across the facility [13]. If species are uniformly distributed and if the horses were similarly attractive, it might be difficult to determine an attraction range. More research is needed to better define the trap attraction range.

To summarize, data from this study indicate that the CDC 1012 and MM-Pro traps are effective at trapping mosquitoes as long as they are not placed in a close competitive situation with a natural host. If adult mosquito trapping is the main technique used for management and surveillance, traps may not provide an accurate representation of the actual mosquito species’ population levels, depending on trap placement. There can be differences in the species composition of mosquitoes caught in traps and those vacuumed directly off a horse. There can be a differential attraction to different horses among mosquitoes. Trap catches may only represent a fraction of the total mosquito population in an area but the traps in our studies do attract the *Cx.* vector species.

Mosquito traps could potentially attract more mosquitoes if host odors could be used as an attractant. Unfortunately, efficacious equine host odor blends have not yet been synthesized, and our attempt to demonstrate equine host odor effects was unsuccessful because of a design flaw. However, more research should be conducted on the subject. Hall et al. [28] isolated octenol, which is currently used as a mosquito attractant [29]. However, horses are attracting mosquito species that are attracted to and repelled by various commercial mosquito attractants, including octenol [13]. At the HTU, large numbers of mosquitoes were feeding on the horses. If the traps are being out-competed by a natural host, then efficacious trap placement and an adequate number of traps are important to effectively manage adult mosquito populations around horses. The attractive distance of a natural host and mosquito traps needs to be investigated and better defined. This could provide personnel working in mosquito management and mosquito trap manufacturing with beneficial data to make recommendations about trap placement, e.g., inside paddocks protected by electric fence [30], to increase the numbers of mosquitoes captured.

## Figures and Tables

**Table 1:** Mean (±SE) and total numbers of mosquitoes captured per 20 min trapping period in the horse barn with a CDC 1012 trap from 19 July–21 August 2004 at the UF Horse Teaching Unit, Gainesville, FL, USA, showing the effect when a horse is placed in the stall adjacent to the trap during period 2 (*n* = 6).

	Horse Present in Period 2		No Horse Present	
Periods	$\bar{X}$ (±SE)	∑	$\bar{X}$ (±SE)	∑
1	51.0 (±13.1) a	306	54.2 (±6.8) e	325
2	6.3 (±2.4) c	38	81.3 (±9.1) d	488
3	33.8 (±5.5) ab	203	52.3 (±10.4) e	314
4	21.2 (±2.1) bc	127	32.7 (±5.9) f	196
5	11.5 (±1.7) bc	69	30.5 (±6.2) f	183

Means in the columns followed by the same letter are not significantly different (*p* = 0.05; Tukey’s Studentized Range Test; SAS 2001).

**Table 2:** Mean (±SE) and total numbers of mosquitoes captured per 20 min trapping period near a feeding slip stall with an MM-Pro trap from 1–21 October 2004 at the UF Horse Teaching Unit, Gainesville, FL, USA, showing the effect when a horse is placed in the stall adjacent to the trap during period 2 (*n* = 6).

	Horse Present in Period 2		No Horse Present	
Periods	$\bar{X}$ (±SE)	∑	$\bar{X}$ (±SE)	∑
1	163.5 (±40.3) a	982	103.7 (±21.1) c	622
2	56.8 (±21.0) b	341	129.8 (±32.9) c	779
3	157.7 (±53.7) a	946	86.2 (±19.1) cd	517
4	79.3 (±15.6) b	476	50.5 (±8.0) d	303
5	57.0 (±14.5) b	341	37.7 (±7.7) d	226

Means in the columns followed by the same letter are not significantly different (*p* = 0.05; Tukey’s Studentized Range Test; SAS 2001). Because it was not possible to conduct treatments in pairs on the same nights, data between treatments were not compared statistically.

**Table 3:** Mean (±SE) and total numbers of mosquitoes captured per 20 min trapping period in the horse barn with a CO_2_-baited CDC 1012 trap from 27 August–24 September 2004 at the UF Horse Teaching Unit, Gainesville, FL, USA, showing the effect when a Paint gelding horse was kept in the stall adjacent to the trap during periods 1–3, and horse odors vacuumed from the horse were added to the trap airstream in period 2.

Treatment 1 (*n* = 6) ^a^			Treatment 2 (*n* = 4)	
Periods	$\bar{X}$ (±SE)	∑	$\bar{X}$ (±SE)	∑
1	34.7 ± 1.9 b	208	111.5 ± 11.1 d	446
2	41.5 ± 2.1 b	249	153.0 ± 12.9 e	612
3	28.8 ± 2.6 b	173	87.3 ± 10.1 c	349
4	79.8 ± 7.6 a	479	66.0 ± 7.7 c	264

Means in the columns followed by the same letter are not significantly different (*p* = 0.05; Tukey’s Studentized Range Test; SAS 2001). Because it was not possible to conduct treatments in pairs on the same nights, data between treatments were not compared statistically. ^a^ Treatment 1 = CO_2_-baited CDC 1012 trap always present, horse present in periods 1–3, not present in period 4 (*n* = 6). Treatment 2 = CO_2_-baited CDC 1012 trap always present, horse never present (*n* = 4).

**Table 4:** Percentage and total number of mosquito species trapped at each CDC 1012 trap position during the location profile study conducted on 13 July–26 August 2004, at the UF Horse Teaching Unit, Gainesville, FL, USA. The highest percentage of species captured at each position is highlighted.

Trap Positions			
Species	1 (*n* = 10)	2 (*n* = 8)	3 (*n* = 9)
*Cq. perturbans*	7.6	8.6	7.4
*Cx. nigripalpus*	45.5	11.4	12.2
*Cx. erraticus*	3.3	1.7	3.4
*Cx. salinarius*	11.5	6.2	5.0
*Ma. titillans*	30.0	71.2	71.5
Other ^1^	2.1	0.9	0.5
Total numbers trapped	1134	4249	1801

^1^ Species representing < 1% of the total catch: *Ps. ferox* (von Humbolt), *Ps. cyanescens* (Coquillett), *Ae. vexans* (Meigen), and *Ma. dyari* (Belkin, Heinemann and Page).

**Table 5:** Percentage of mosquito species and total number and number/h/horse of mosquitoes vacuumed from horses during two 2004 studies at the UF Horse Teaching Unit, Gainesville, FL, USA (*n* = 6). Species captured in the highest percentage by the Paint gelding and Appaloosa mare together and separately in the October study are highlighted.

Study Dates				
15 June–12 July		4–22 October		
Mosquito Species	Paint Gelding	Paint Gelding + Appaloosa Mare	Appaloosa Mare	Paint Gelding
*An. crucians* ^1^	0.3	0.8	0.7	1.1
*An. quadrimaculatus* ^2^	0.0	2.3	1.9	3.3
*Cq. perturbans*	39.4	1.8	1.5	2.3
*Cx. erraticus*	2.2	5.3	6.4	3.1
*Cx. nigripalpus*	9.4	41.7	50.0	25.0
*Cx. salinarius*	36.9	2.4	2.4	2.4
*Ma. titillans*	11.8	27.6	21.1	40.6
*Oc. infirmatus* ^3^	0.0	0.1	0.1	0.3
*Ps. ciliate* ^4^	0.0	0.3	0.3	0.3
*Ps. columbiae*	0.0	17.4	15.5	21.1
*Ps. ferox*	0.0	0.3	0.3	0.4
Total catch	1946	7197	4810	2387
Catch/h/horse	324	359	480	238

Collection period for 15 June–12 July study = 1 h; for 4–22 October = 1 h 40 min. ^1^ *Anopheles crucians* Wiedemann; ^2^ *An. quadrimaculatus* Say; ^3^ *Ochlerotatus infirmatus* Dyar and Knab; ^4^ *Ps. ciliata* (Fabricius).

**Table 6:** Mean (±SE) and total number of mosquitoes vacuumed from each horse when one horse is separated from the other horse by the designated distance; 4–22 October 2004 study, UF Horse Teaching Unit, Gainesville, FL, USA.

Appaloosa Mare			Paint Gelding		
Distance (m)	Mean (±SE)	Total Catch	Mean (±SE)	Total Catch	*n*
3.1	172.3 (±35.8) a	1034	104.0 (±10.1) b	624	6
6.2	202.0 (±51.0) a	1212	92.8 (±18.6) b	557	6
9.1	175.2 (±34.7) a	1051	93.3 (±11.0) b	560	6
12.2	159.8 (±35.3) a	959	73.3 (±10.2) b	440	6
20.4	184.7 (±22.3) a	554	68.8 (±13.8) b	206	3

Means in the rows followed by the same letter are not significantly different (*p* = 0.05; Tukey’s Studentized Range Test; SAS 2001).

## Data Availability

Data sets were quite small and have been lost over time.
